# A stiff and straight back preoperatively is associated with a good outcome 2 years after lumbar disc surgery: A prospective study of 80 patients

**DOI:** 10.3109/17453670903316785

**Published:** 2009-10-01

**Authors:** Anders Lundin, Anders Magnuson, Olle Nilsson

**Affiliations:** ^1^Department of Orthopedics, Örebro University HospitalÖrebroSweden; ^2^Unit of Statistics and Epidemiology, Örebro University HospitalÖrebroSweden; ^3^Department of Orhopedics, Uppsala University HospitalUppsalaSweden

## Abstract

**Background and purpose** The degree of lumbar lordosis and reduced lumbar mobility are regarded as important clinical features in patients with low back pain, and in lumbar disc herniation A more stiff back preoperatively in a proportion of patients has been shown to be associated with sequestered disc herniation. The main aim of this study was to investigate whether there was any correlation between lumbar lordosis and flexion on the one hand in patients with lumbar disc herniation who were scheduled for surgery, and postoperative pain and disability on the other. Our second aim was to determine the patterns of postoperative improvement in pain, perceived disability, and flexion/lordosis for 2 years after surgery.

**Methods** Pain (VAS), disability (DRI), lumbar flexion and lordosis (Debrunner's kyfometer) were measured pre- and postoperatively in 80 patients who underwent microscopic lumbar disc surgery.

**Results** Patients with preoperative hyperlordosis had more severe pain and more disability postoperatively than patients with hypolordosis. The level of pain did not change much from 2–6 weeks postoperatively until 2 years, while the perceived disability did not reach a steady state until 6 months after surgery.

**Interpretation** Patients with a stiff and flat back have a good prognosis after lumbar disc surgery, and in most cases the pain will reach the 2-year level during the first 2–6 weeks, while the physical restoration measured by the lumbar flexion and lordosis, and the perceived disability, will continue to improve over the first 6 months after surgery.

## Introduction

The degree of lumbar lordosis and reduced lumbar mobility are regarded as important clinical features in patients with low back pain, and in lumbar disc herniation. For example, the range of motion of the lumbar spine has been shown to be predictive of the type of disc herniation found at surgery. Those with a stiff back preoperatively was associated to have a sequestered disc herniation. Also, a subset of patients with sequestrated hernias, who had a good clinical outcome, increased both their lumbar flexion and their lordosis postoperatively (Vucetic and Svensson 1996). In addition, a positive correlation between the degree of lordosis and lumbar sagittal movement has been described in young female gymnasts (Ohlen et al. 1989b). Thus, according to these findings the degree of lumbar lordosis and flexion preoperatively in lumbar disc herniation could be used as one of several clinical signs for prediction of the outcome of surgery. Another important task is to determine at which time point we can expect to be able to predict the end result after lumbar disc surgery in order to find the patients who might benefit from additional rehabilitation measures. 2 months after surgery has been proposed to be a sufficient time period to allow prediction of the result at one year, but the results were not fully conclusive (Hakkinen et al. 2003).

The main aim of this study was to investigate any correlation between the degree of lumbar lordosis/flexion in patients with lumbar disc herniation who were scheduled for surgery, and postoperative pain and perceived disability. Our second aim was to determine at which time point after surgery the 2-year results can be predicted.

## Patients and methods

The study population consisted of 80 patients (44 men) who underwent microscopic lumbar disc surgery to determine the effect of peroperatively administered corticosteroids from October 1994 through November 1998 (Lundin et al. 2003). The inclusion criteria were: age between 18 and 65 years, no history of drug abuse, no earlier surgery at the affected level, duration of sciatica less than 1 year, MRI verified L3-4, L4-5, or L5-S1 lumbar disc herniation and clinical presentation of sciatica consistent with MRI findings. All the patients underwent a clinical examination, including measurement of lumbar flexion and lordosis using Debrunner's kyfometer (Ohlen et al. 1989a) on the day before surgery and postoperatively after 2 weeks, 6 weeks, 3 months, 6 months, 12 months, and 2 years.

The mean age was 42 (18–65) years. The median duration of sciatica was 4.5 (0–12) months and the median duration of back pain was 5 (1–24) months.

Pain was assessed by the patients on a visual-analog scale ranging from 0 (no pain) to 100 (worst pain possible) documenting pain just now (VAS-N) and worst pain during last week (VAS-W). Impairment was assessed with the Disability Rating Index (DRI) (Salen et al. 1994), which consists of 12 items concerning physical function in a self-assessment form. This questionnaire has been compared to the Oswestry scale by Grotle et al. (2004) and was found to be equal in measuring changes in functional status.

### Kyphometer

We used the Debrunner's kyphometer (Protek AG, Bern Switzerland); this has been tested by Öhlen et al. (1989a), who found a mean standard deviation of 2.7° for lordosis and 3.6° for lumbar flexion in their analyses of variance. We measured the lumbar lordosis between T11-T12 and S1-S2. The degree of lordosis is read directly on the instrument. The patient was told to stand relaxed and then to bend forward as much as possible. The degree of flexion is the difference between the value at the starting position and that at the maximum forward bending, in absolute numbers.

We dichotomized the patients based on lumbar flexion (by the median value) into hypo- and hyperflexion, and in the same manner based on lordosis into hypo- and hyperlordosis. The median was used as a cut-off value: a patient with a more negative value than the median of lordosis was categorized arbitrary as having a hyperlordosis, and a patient with a more positive value (closer to zero) was categorized as having a hypolordosis.

### Statistics

Severe pain was defined as VAS-W or VAS-N of more than 54 mm on the VAS scale according to the study of Collins et al.(1997). DRI was categorized at 40. We considered this cut-off point to be reasonable, based on the results from the study of Salen et al. (1994), who found that all healthy subjects scored below 40, and that patients with hip and knee arthrosis had a median score above 40. The main aim was to determine whether hyper-/hypoflexion and hyper-/hypolordosis were associated with pain postoperatively (VAS-N and VAS-W) and disability (DRI). To control for the fact that this study population consisted of patients randomized to treatment with corticosteroids or placebo, with better outcomes in the corticosteroid group (Lundin et al. 2003), we used a type of regression model called generalized estimating equation (GEE) with logit link function and binomial error term. As independent variables we used corticosteroid group (yes/no), severe pain or disability preoperatively (DRI) rating over 40 (yes/no), because preoperative pain and disability could influence the end results. We also adjusted for sex and age, since women generally assess higher levels on VAS (Hakkinen et al. 2007, Stromqvist et al. 2008), and higher levels of pain have been reported at higher age (Miranda et al. 2002). Due to repeated measurements on each patient (at 2 weeks, 6 weeks, 3 months, 6 months, 1 year, and 2 years), an autoregressive correlation structure was used, which means that the correlation is highest for adjacent time points and decreases when time points are further apart. The computation was done using the GENMOD procedure in SAS software version 8.02.

## Results

Preoperatively, the mean pain measured as VAS-N was 51 (SD 25) and as VAS-W it was 82 (SD 20). The mean DRI was 69 (SD 17). 91% of patients had a positive Laségue's sign and 25% had positive crossed Laségue's sign.

There was one drop-out because of such severe pain preoperatively that the patient could not perform standing and flexion maneuvers. The median of lordosis in the remaining 79 patients was –20º (–42 to 30).

The median of flexion in the same 79 patients was 24º (0–76). Patients with higher values than the median were categorized arbitrary as having a hyperflexion, and patients with lower values were categorized as having a hypoflexion.

Preoperatively, a somewhat higher proportion of patients with hyperlordosis had severe pain (51% VAS-N) compared to patients with hypolordosis (34% VAS-N), while VAS-W and DRI were about the same (Tables 1–3). Similarly, the patients with hypoflexion had more severe pain (50% VAS-N) than the hyperflexion group (36% VAS-N). There was also a somewhat higher proportion of hypoflexion patients with DRI > 40 (100%, as opposed to 87% in the hyperflexion group), whereas VAS-W scores were similar in both groups.

**Table 1.** Numbers of patients with severe pain measured as pain right now (VAS-N > 54) and results from generalized estimating equation (GEE) models expressed with odds ratios (ORs) supplemented with 95% confidence intervals (CIs). An odds ratio of > 1 indicates more pain

	Preop.	2 weeks	6 weeks	3 months	6 months	1 year	2 years	OR **^a^** (95% CI)	p-value
*All* (n = 79)	34	2	4	4 **^c^**	2 **^c^**	5 **^c^**	5 **^c^**		
Hypoflexion (n = 40)	20	1	2	2	1 **^c^**	2 **^c^**	2 **^c^**	reference	
Hyperflexion (n = 39)	14	1	2	2 **^c^**	1	3	3	1.25 (0.27–5.7)	0.8
Hyperlordosis (n = 41)	21	2	2	4 **^c^**	1	4 **^c^**	4	reference	
Hypolordosis (n = 38)	13	0	2	0	1 **^c^**	1	1 **^c^**	0.60 (0.15–2.4)	0.5
*Only patients with hypoflexion*									
Hyperlordosis (n = 16)	16	1	2	2	1	2 **^c^**	2	reference	
Hypolordosis (n = 24)	21	0	0	0	0 **^c^**	0	0 **^c^**	Not possible to estimate **^b^**	
*Only patients with hyperflexion*									
Hyperlordosis (n = 25)	21	1	0	2 **^c^**	0	2	2	reference	
Hypolordosis (n = 14)	13	0	2	0	1	1	1	2.90 (0.85–9.9)	0.2
**^a^** Adjusted for corticosteroid treatment, preoperative pain, time, sex, and age.										**^b^** Not possible to estimate because none of the patients with hypolordosis had severe pain postoperatively.										**^c^** 1 patient is missing.									

**Table 2.** Numbers of patients with severe pain measured as worst pain last week (VAS-W > 54) and results from generalized estimating equation (GEE) models expressed with odds ratios (ORs) supplemented with 95% confidence intervals (CIs). An odds ratio of > 1 indicates more pain

	Preop.	2 weeks	6 weeks	3 months	6 months	1 year	2 years	OR **^a^** (95% CI)	p-value
*All* (n = 79)	71	14 **^c^**	8	10 **^c^**	11 **^c^**	13 **^c^**	12 **^c^**		
Hypoflexion (n = 40)	37	6	5	5	5 **^c^**	5 **^c^**	5 **^c^**	reference	
Hyperflexion (n = 39)	34	8 **^c^**	3	5 **^c^**	6	8	7	1.1 (0.45–2.9)	0.8
Hyperlordosis (n = 41)	37	10 **^c^**	4	8 **^c^**	9	8 **^c^**	9	reference	
Hypolordosis (n = 38)	34	4	4	2	2 **^c^**	5	3 **^c^**	0.42 (0.16–1.1)	0.07
*Only patients with hypoflexion*									
Hyperlordosis (n = 16)	16	5	3	5	5	4 **^c^**	5 **^b^**	reference	
Hypolordosis (n = 24)	21	1	2	0	0 **^c^**	1	0 **^c^**	0.06 (0.01–0.27)	0.003
*Only patients with hyperflexion*									
Hyperlordosis (n = 25)	21	5 **^c^**	1	3 **^c^**	4	4	4	reference	
Hypolordosis (n = 14)	13	3	2	2	2	4	3	1.2 (0.37–4.2)	0.8
**^a^** Adjusted for corticosteroid treatment, preoperative pain, time, sex, and age.										**^b^** Adjusted for corticosteroid treatment, time, sex, and age but not for preoperative pain because of concordance, all patients had severe pain preoperatively.										**^c^** 1 patient is missing.									

**Table 3.** Numbers of patients with DRI > 40 and results from generalized estimating equation (GEE) models expressed with odds ratios (ORs) supplemented with 95% confidence intervals (CIs). An odds ratio of > 1 indicates more perceived disability

	Preop.	2 weeks	6 weeks	3 months	6 months	1 year	2 years	OR **^a^** (95% CI)	p-value
*All* (n = 79)	74	66	37	19 **^c^**	11 **^c^**	9 **^c^**	10 **^c^**		
Hypoflexion (n = 40)	40	33	19	10	4 **^c^**	4 **^c^**	4 **^c^**	reference	
Hyperflexion (n = 39)	34	33	18	9 **^c^**	7	5	6	1.3 (0.57–3.0)	0.5
Hyperlordosis (n = 41)	37	36	23	13 **^c^**	8	7 **^c^**	8	reference	
Hypolordosis (n = 38)	37	30	14	6	3 **^c^**	2	2 **^c^**	0.37 (0.16–0.86)	0.02
*Only patients with hypoflexion*									
Hyperlordosis (n = 16)	16	15	13	7	4	3 **^c^**	4	reference	
Hypolordosis (n = 24)	24	18	6	3	0 **^c^**	1	0 **^c^**	0.11 (0.04–0.35) **^c^**	< 0.001
*Only patients with hyperflexion*									
Hyperlordosis (n = 25)	21	21	10	6 **^c^**	4	4	4	reference	
Hypolordosis (n = 14)	13	12	8	3	3	1	2	1.3 (0.38–4.1)	0.7
**^a^** Adjusted for corticosteroid treatment, preoperative pain, time, sex and age.										**^b^** Adjusted for corticosteroid treatment and time but not preoperative disability because of concordance, all patients had DRI > 40 preoperatively.										**^c^** 1 patient is missing.									

### Vas N

Neither the preoperative degree of flexion (p = 0.8) nor lordosis (p = 0.5) was statistically significantly associated with severe pain postoperatively (Table 1). The statistical interaction between flexion and lordosis could not be estimated due to there being too few observations. Stratification of flexion did not show any statistically significant associations between degree of lordosis and pain postoperatively, either in patients with hypoflexion or in patients with hyperflexion.

### Vas W

There was a tendency for patients with hypolordosis preoperatively to have less severe pain postoperatively than patients with hyperlordosis (OR 0.42, 95% CI: 0.16–1.1; p = 0.07) (Table 2). The difference was fairly homogenous from 2 weeks onward, except for the 6-week time point. Patients with hyperflexion preoperatively had somewhat more pain postoperatively than patients with hypoflexion, but the difference was far from statistically significant (OR 1.1, CI: 0.45–2.9; p = 0.8). The statistical interaction between flexion and lordosis was significant (p = 0.004), meaning that the difference in postoperative pain between patients with hyper- and hypolordosis depended on the patient degree of flexion. With stratification of the analyses of flexion and looking first only at patients with hypoflexion preoperatively, patients with hypolordosis had less pain postoperatively than patients with hyperlordosis (OR 0.06, CI: 0.01–0.27; p = 0.003). Secondly, looking only at patients with hyperflexion preoperatively, no association was found.

### DRI

A lower proportion of patients with preoperative hypolordosis compared to patients with hyperlordosis had DRI > 40 postoperatively (OR 0.37, CI: 0.16–0.86; p = 0.02) (Table 3). No statistically significant association could be found between patients with hyperflexion preoperatively compared to patients with hypoflexion (p = 0.5). The statistical interaction between flexion and lordosis was significant (p = 0.008). Stratifying the analyses of flexion and looking first only at patients with preoperative hypoflexion, a lower proportion of patients with hypolordosis preoperatively had DRI > 40 postoperatively than patients with hyperlordosis (OR 0.11, CI: 0.04–0.35; p < 0.001). Secondly, looking only at patients with preoperative hyperflexion, no association was found.

### Postoperative development of pain, perceived disability, lumbar flexion and lordosis

**Figure 1.**
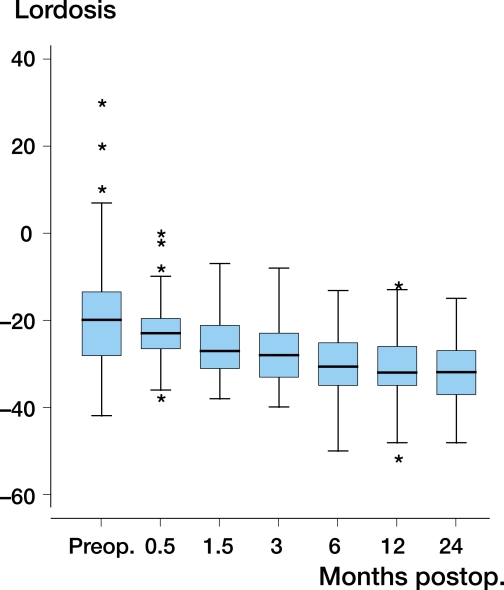
The distribution of lumbar lordosis in all patients (n = 79), measured preoperatively to 2 years postoperatively.

**Figure 2.**
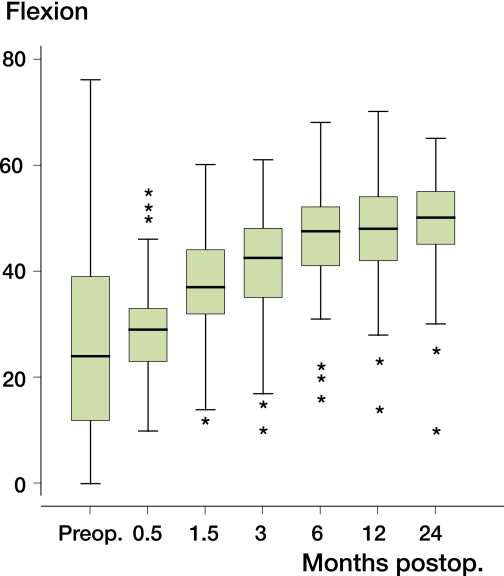
The distribution of lumbar flexion in all patients (n = 79), measured preoperatively to 2 years postoperatively.

It may be concluded that level of pain did not change much between 2–6 weeks postoperatively and 2 years, while the perceived disability did not reach a steady state until 6 months after surgery (Tables 1–3). The pattern of recovery of flexion and lordosis postoperatively was similar to the development of DRI: there was an increment in flexion and lordosis up to 6 months, while the reduction in pain occurred earlier (Figures 1 and 2).

### Complications

In 6 patients, we found the following complications. There was 1 discitis 6 months postoperatively, which was successfully treated with antibiotics. There were 3 relapses of disc herniation (1 of these patients was operated on 10 months after the primary surgery). 1 patient received a herniation at a new level but had no further surgery. 1 patient developed a post-discectomy syndrome and went through a fusion 20 months after the disc surgery. According to the study design, the analyses included all these patients.

## Discussion

We found that a stiff and flat back preoperatively is associated with a good outcome 2 years after lumbar disc surgery. One explanation might be that this lumbal configuration is one way to diminish the mechanical pressure from the herniated disc (Fennell et al. 1996). To achieve this stature, the spinal muscles and the anatomical configuration may be developed in a more adaptable way. These factors could then enhance the postoperative rehabilitation. Certainly, there is not just one clinical predictor of outcome in lumbar disc surgery, but it appears to be of value to determine the lordosis and degree of flexion preoperatively. Combined with other findings, e.g. MRI and Laségue's sign, this could help us to detect patients with a clinical pattern indicative of a good result of disc surgery, and to identify the patients who are at risk of a less favorable outcome. The finding of a positive association between a straight lumbar spine and a positive outcome of surgery is in agreement with the previous findings of an association between lumbar flexion and positive outcome (Astrand et al. 2000), since there is a high correlation between lordosis and flexion. The analysis of these clinical features is facilitated by the fairly large variability in the degree of lordosis and mobility of the lumbar spine preoperatively.

The pattern of reduction in pain after surgery indicates that a steady state will be reached in the period between 2 and 6 weeks, somewhat earlier than shown by Hakkinen et al. (2003). In a later study, however, Hakkinen et al. (2007) found that most of the improvement occurred during the first 6 weeks postoperatively. The patients with severe pain 2 years after surgery could not be identified at the first 2 time points: 2 weeks and 6 weeks. On the other hand, most patients with good improvement at 2 weeks were doing well also at 2 years.

Measurement of pain in 2 ways (VAS-N and VAS-W) did not give identical results, but the tendency was the same. One explanation for this discrepancy might be that VAS-W estimates the pain in a more comprehensive way, similar to DRI, while VAS-N describes the daily variation in pain. In order to analyze pain (VAS) and disability (DRI) as ordinal data, as recommended by some authors (Svensson 2001) we stratified for severe pain (VAS > 54) according to a study by Collins et al. (1997), and for severe disability (DRI > 40) (Salén et al. 1994). This might induce a weakness in the analysis since it is not obvious where to place cut-offs. However, we also performed the analyses with data as continuous numerical values and found that this did not alter the results. Interestingly, the 6 patients with postoperative complications were all recruited from the group with hyperlordosis, which could be one explanation for the inferior outcome in this group. To analyze this further, we excluded these 6 patients in a second analysis, and this did not alter the results of the primary analysis.

We found a pattern of continuous improvement in the degree of flexion and in restitution of lordosis. This finding disagrees somewhat with the results of Mannion et al. (2005) who, despite seeing a good clinical outcome, generally found a more stiff and erect lumbar spine 2 months after lumbar disc surgery compared to preoperative values. One explanation for this discrepancy may be the fact that these patients were older and had a longer duration of back and leg pain than those in our study. Since we measured function and disability at 6 time points after surgery, until 2 years, we could detect an association between the physical improvement and the perceived disability. The restitution of flexion and lordosis followed a pattern similar to that of DRI, which was slower than the decrease in pain. To some extent, this slower increase in rate of recovery of function can be attributed to the advice the patients are given after surgery not to indulge in vigorous rehabilitation. However, the restitution of lordosis and regain in lumbar flexion could also be an expression of the effects of the herniation itself on these functions and not only be secondary to the reduced pain, since we found that the improvement in pain preceded the perceived disability and the restoration of the lordosis and flexion. These findings do not support the results of Vucetic and Svensson (1996) who considered the range of lumbar movement to be caused by the perceived pain.

One weakness of our study was that the data was collected from a study designed for another purpose. Despite this we believe that it is of clinical interest to describe the pattern of the patients suffering from disc herniation.
